# Association between Circulating Endothelial Cells and Carotid Atherosclerosis in Patients Receiving Maintenance Hemodialysis

**DOI:** 10.1155/2014/753759

**Published:** 2014-03-20

**Authors:** Zhang Kun-ying, Liu Hui-lan, Duan Xiao-feng, Li Guo-gang

**Affiliations:** ^1^Department of Nephrology, Weifang People's Hospital, Weifang, Shandong 261041, China; ^2^Department of Nephrology, Fuxing Hospital, Capital University of Medical Science, Beijing, China

## Abstract

Accelerated atherosclerosis is the major cause of mortality in maintenance hemodialysis (MHD) patients, and endothelial injury associated with MHD might contribute strongly to pathogenesis. The current study was designed to explore possible associations between circulating endothelial cells (CECs) and intima-media thickness of common carotid artery (CCA-IMT) as an indicator of carotid atherosclerosis. Sixty-two MHD patients and 26 age- and sex-matched healthy volunteers were recruited. The number of CECs was determined in peripheral blood using multiparametric flow cytometry. CCA-IMT and presence of plaques in the common carotid arteries were assessed with ultrasound. Laboratory tests results and the demographics were recorded. The finding indicated that numbers of CECs were higher in patients before hemodialysis (predialysis) compared with numbers in controls (*P* = 0.045). CCA-IMT was also significantly higher in patients than in controls (*P* < 0.01). A positive relationship was observed between predialysis CECs numbers and CCA-IMT (*P* < 0.01) in MHD patients. In multiple linear regression analysis, the relationship between the predialysis CECs level and CCA-IMT remained the same even if adjusting for confounding effects. Accordingly, the investigation indicates that the CECs level is positively associated with CCA-IMT in our hemodialysis patients. CECs might be an important marker to the severity of carotid atherosclerosis in MHD patients.

## 1. Introduction

Accelerated atherosclerosis tends to be advanced in patients on maintenance hemodialysis (MHD), and a major cause of mortality among these patients is atherosclerotic cardiovascular disease [1, 2]. Numerous factors including uremic toxins, hypertension, obesity, hyperlipidemia, and increased oxidative stress have been found to be strongly associated with atherosclerosis in MHD patients [3, 4]. There is growing evidence that increased intima-media thickness of the carotid artery (CA-IMT) is considered as a confirmed and accepted indicator of atherosclerotic changes [5, 6], and several studies have indicated increased CA-IMT can also predict cardiovascular mortality in hemodialysis patients [7, 8].

Recent evidence demonstrated that endothelial dysfunction may play a crucial role in initiation and pathogenesis of atherosclerosis [9]. Endothelial damage can be assessed in several ways, such as by physiological techniques as flow mediated dilatation [10], and by the measurement of soluble markers as cell adhesion molecules and von Willebrand factor in the peripheral blood [11, 12]. In recent years, circulating endothelial cells (CECs) have been recognized as a potential marker for endothelial state [13]. The number of CECs increased markedly in MHD patients [14], and increased numbers have been shown to be important predictors of long-term cardiovascular events in MHD patients [15].

Both increased CA-IMT and increased CECs level were associated with high cardiovascular mortality in hemodialysis patients [7, 8, 15], but the relationship between CECs and carotid atherosclerosis in these patients is still limited. We hypothesized that CECs level not only reflects endothelial dysfunction but also is related to the severity of carotid atherosclerosis in MHD patients. Accordingly, we designed this study to investigate the relationship between CECs and intima-media thickness of common carotid artery (CCA-IMT).

## 2. Methods

### 2.1. Study Patients

In the cross-sectional study, we designed to explore the relationship between CECs and carotid atherosclerosis in MHD patients. Sixty-two patients (29 males, 33 females) undergoing long-term hemodialysis were recruited in a dialysis center in Beijing, China. The inclusion criteria were (1) more than 18 years old; (2) in stable condition, and on maintenance hemodialysis for at least 6 months; Kt/V > 1.2. The exclusion criteria were (1) central catheter insertion or any invasive procedure during the month before blood collection; (2) signs or symptoms of any kind of chronic or acute infection within one month before blood collection; (3) diagnosis of cancer; (4) positive human immunodeficiency virus serology; and (5) hepatitis B or C infection.

All patients were treated with conventional hemodialysis (HD) and were dialyzed three times per week for 5 hours per session with a blood flow of 250–300 mL/min and a dialysate flow of 500 mL/min. No patient reused dialyzer membranes. Overall, 58.7% of patients took antihypertensive medication including calcium channel blockers (CCB, 37.1%), angiotensin-converting enzyme inhibitors (ACEI, 15.6%), and alpha or beta receptor antagonists (24.3%). Statins were used for dyslipidemia by 15.6%. No patient took steroids. Patients were studied without washout of regular medications.

Twenty-six age- and sex-matched healthy individuals (12 males, 14 females) were enrolled as controls. Controls were recruited from hospital staff and their families.

MHD patients were classified by CCA-IMT level into three subgroups according to previous recommendation [16]: group A, or normal IMT group, had IMT < 0.8 mm (*N* = 24); group B, or abnormal IMT group, had IMT level between 0.8–1.1 mm (*N* = 23), and group C, or thickened IMT group, had IMT ≥ 1.1 mm (*N* = 15).

This study was approved by the local ethics committee and each subject gave an informed consent prior to participation.

### 2.2. Sample Collection and Laboratory Procedures

Blood samples for CECs determination were drawn from the arteriovenous fistula just before dialysis session in MHD patients and from a forearm vein in controls after discharge of the first 3 mL of blood. All subjects were in fasting condition. Blood was collected into ethylene diamine tetra-acetic acid (EDTA) tube. Anticoagulated blood samples were kept at 4 degree centigrade and then were transported to the laboratory for flow cytometry within six hours.

Peripheral whole blood cells were prepared by a lyse/no-wash procedure using Trucount tubes (Becton Dickinson, San Jose, CA) with 51500 beads in each tube and were then evaluated by FACSCalibur flow cytometry (Becton Dickinson, San Jose, CA). This procedure minimizes cell loss, making it possible to measure all circulating endothelial cells in a blood sample. A multistep manual technique was used to detect and quantify circulating endothelial cells. Assessment of a minimum of 100,000 cells/100 *μ*L peripheral blood was considered informative. A new panel of monoclonal antibodies (anti-CD146 and anti-CD3) was used to detect the cells. The antibodies were conjugated with phycoerythrin (PE-CD146, Becton Dickinson Pharmingen, San Diego, CA) and peridinin chlorophy II protein (PerCP-CD3, Becton Dickinson, San Jose, CA). Cells were identified as circulating endothelial cells if they were CD3^−^CD146^+^. Nonviable cells, platelets, debris, and nonspecific binding were excluded from analyses by isotopic control and consecutive gating. Flow cytometry data were analyzed with CellQuest software.

Blood urea nitrogen (BUN), serum creatinine (Scr), albumin, calcium, phosphate, high-density lipoprotein cholesterol (HDL-c), low-density lipoprotein cholesterol (LDL-c), and hemoglobin were measured by standard laboratory techniques using an automatic analyzer. The demographics were recorded.

### 2.3. Evaluation of Common Carotid Artery Intima-Media Thickness

CCA-IMT was measured by high-resolution B-mode ultrasonography using a real-time ultrasonograph with a 4.0–6.0 MHz in-line Sectascanner (Acuson Sequoia 512, Mountain View, CA). Ultrasonographic study of patients was performed after dialysis within one week of blood sampling. Bilateral investigation was done by one expert radiologist who was unaware of clinical and laboratory data. Subjects were in the supine position with head slightly turned from echography at measurement. Measurement of IMT was performed 0.5 to 1.0 cm proximal to the beginning of the carotid bulb on the wall of the CCA. IMT was defined as the distance between the leading edge of the lumen-intima echo of the near wall and the leading edge of the media-adventitia echo. IMT was measured on longitudinal views of the far wall of the distal segment of the CCA. Mean values of IMT were calculated from at least three measurements for each artery. The average of the obtained values was taken as IMT; it was considered abnormal when it exceeded 0.8 mm. Evaluation for carotid plaques, which were defined either as faint grey echoes (soft plaques) or bright white echoes (calcified plaques) protruding into the arterial lumen in the presence of IMT > 1 mm [17], was performed for the common, internal, and external carotid arteries bilaterally. Plaque thickness was measured in a suitable longitudinal or transverse view.

### 2.4. Statistical Analyses

The relationships between CECs and each of systolic blood pressure, age, body mass index, HDL-c, hemoglobin, and albumin levels were tested using Pearson correlation analysis. Levels of each of CECs, age, body mass index, systolic blood pressure, diastolic blood pressure HDL-c, albumin, calcium, and hemoglobin were compared between MHD patients and controls using* t*-test. The above parameters were also compared using one-way analysis of variance (1-way-ANOVA) among groups A, B, and C in MHD patients. Using CCA-IMT as outcome and CECs as predictor, one linear regression model was constructed to explore the relationship between CECs and CCA-IMT after gender, age, primary diseases, dialysis vintage, and other confounding effects were adjusted. Median, range, percentiles, and Kruskal Wallis Test were used for nonnormally distributed variables. Numeric parameters were assessed by *χ* ^2^ test. Statistical analysis was performed using SPSS version 11.5 (SPSS, Inc., Chicago, IL, USA). *P* values <0.05 were considered as statistically significant.

## 3. Results

### 3.1. Baseline Characteristics of Subjects

The characteristics of all the included patients were listed in Table 1. There were 62 MHD patients, with a mean age of 61.8 ± 12.9 years and a mean dialysis vintage of 57.2 ± 45.4 months. And there were 26 healthy individuals with a mean age of 58.3 ± 12.4 years.

Primary renal diseases were chronic glomerulonephritis (*n* = 14), interstitial nephropathy (*n* = 11), diabetic nephropathy (*n* = 18), hypertensive nephrosclerosis (*n* = 11), polycystic disease (*n* = 3), and others (*n* = 5).

Compared with controls, values for HDL-c, albumin, calcium, and hemoglobin were significantly decreased in patients, confirming a metabolic difference between groups. In addition, systolic blood pressure and phosphate were elevated in patients; age, gender, body mass index, and diastolic blood pressure were similar between groups (Table 1).

### 3.2. Circulating Endothelial Cells and Clinical Parameters

**Figure 1:**
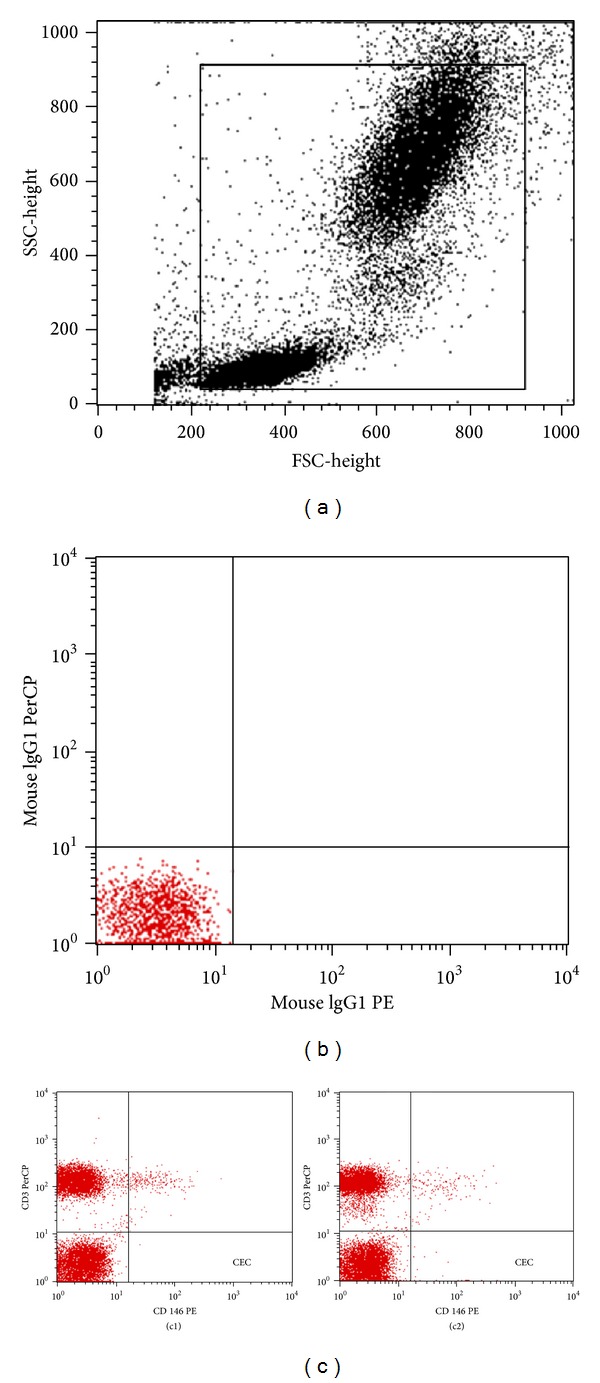
Gating strategy for identification and quantification of circulating endothelial cells. (a) Gate enclosing all cells for further analysis. (b) Platelets, debris, and nonspecific binding were excluded from analysis by isotopic control. (c) Cells from the FS/SS plot examined for expression of CD3^dim⁡^/CD146^bright^. These CD3^dim⁡^/CD146^bright^ cells are identified as circulating endothelial cells (lower right quadrant) (C1, distribution of cells in control subjects; C2, distribution of cells in patients).

We measured the number of CECs using multiparametric flow cytometry (FCM, Figure 1). When compared with control data (*n* = 26, mean 108.7 ± 21.9 cells/mL), CECs level was higher in patients before dialysis (*n* = 62; mean 147.4 ± 95.8 cells/mL; *P* = 0.045). Among patients, there was a significant positive relationship between predialysis cell counts and systolic blood pressure (Pearson *r* = 0.307, *P* = 0.015), age (Pearson *r* = 0.353, *P* = 0.005), and body mass index (Pearson *r* = 0.439, *P* < 0.001). No significant association was found between CECs level and each of calcium, phosphate, hemoglobin, or albumin.

No significant difference was found in CECs level between males (131.6 ± 87.9 cells/mL) and females (161.4 ± 101.6 cells/mL, *P* = 0.225), or no significant difference was found in CECs level among primary diseases (data was not shown).

### 3.3. CCA-IMT and Atherosclerotic Plaques

Compared with controls, patients had significantly increased CCA-IMT (0.7 ± 0.1 mm versus 0.9 ± 0.4 mm, *P* < 0.01). CCA-IMT was elevated (>0.8 mm) in 38 (61.3%) patients and 4 (15.4%) controls. Atherosclerotic plaques were detected in 48 (77.4%) patients and 2 (7.7%) controls. The difference in plaque prevalence between groups was significant (*P* < 0.001). Number of plaques ranged from 0 to 2 in controls and from 0 to 14 in patients.

There was a positive relationship between CCA-IMT and age (Pearson *r* = 0.495, *P* < 0.01). Compared with group A, groups B and C had increased CCA-IMT (*P* < 0.01 and *P* < 0.01, resp.); compared with group B, group C had increased CCA-IMT (*P* < 0.01, Table 2).

### 3.4. Association between CECs Level and CCA-IMT

There was a positive relationship between predialysis CECs level and CCA-IMT (Pearson *r* = 0.487, *P* < 0.01) in MHD patients.

Compared with groups A and B, group C had increased CECs level (*P* = 0.002 and *P* = 0.010, resp.; Table 2), there was no significant difference between groups A and B (*P* = 0.480, Table 2). The characteristics of gender, dialysis vintage, body mass index, systolic blood pressure, diastolic blood pressure, and albumin were comparable among all three subgroups. However, patients in groups B and C were older than patients in group A (*P* < 0.01 and *P* < 0.01, resp.); and when compared with group A, group C had lower serum creatinine concentrations (*P* = 0.006) (Table 2).

CECs level remained significantly associated with CCA-IMT after adjusting for age, gender, primary diseases, dialysis vintage, systolic blood pressure, and body mass index in multiple linear regression analysis (*P* = 0.001, Table 3).

## 4. Discussion

In the present study, we found that predialysis CECs level, CCA-IMT and prevalence of plaque were significantly increased in MHD patients compared with age- and sex-matched controls. Furthermore, we also found that predialysis CECs level was closely related to CCA-IMT, as an indicator of carotid atherosclerosis, even after confounding effects were adjusted.

Atherosclerosis is prevalent in MHD patients; these patients are known to have an escalating increment of carotid IMT compared with age- and gender-matched subjects [18–20]. In accordance with previous reports, the present study demonstrated that CCA-IMT and prevalence of plaque were significantly increased in MHD patients compared with controls.

Endothelial dysfunction is recognized as the initial step in the atherosclerotic process. Smooth muscle cell proliferation and intimal thickening can be stimulated experimentally by disrupting the endothelium [21]. Some factors, such as antiendothelial cell antibodies [22], the disturbed flow-induced p53 and ERK5 SUMOylation [23], increased expression of cell adhesion molecules, altered nitric oxide metabolism, and increased inflammation [24], affect endothelial cells in multiple ways. Endothelial dysfunction results in increased monocyte infiltration and their differentiation into macrophages, which take up modified cholesterol-rich lipoproteins to form “foam cells” [25]. This may lead to the formation of atherosclerotic lesions and the increased circulating endothelial cells (CECs).

In recent years, CECs have been developed as a sensitive and specific marker for assessing endothelial damage [13, 26, 27]. The number of CECs is very low in healthy individuals, about 0–12/mL blood [28, 29]. Increased levels of CECs have been observed in a number of diseases with widespread vascular damage, as recently demonstrated in patients with sickle cell anemia [13, 30], ANCA-associated small-vessel vasculitis [29], acute myocardial infarction, unstable angina [31], and severe peripheral artery disease [32]. Moreover, elevated levels of CECs also have a good relationship with the severity of diseases [31, 33]. CECs levels are higher in patients with acute illness than those in recovery phase or in clinical remission of the disease. For example, the level of CECs is higher in patients with acute myocardial infarction than in patients with angina [31]. Woywodt et al. found high level of CECs was detected in patients with active ANCA-associated small vessel vasculitis, whereas the cell numbers declined progressively during the successful therapy [29]. Koç et al. reported that increased CECs level is also found in MHD patients, which paralleled our results. The present study indicated that the CECs level before dialysis was significantly higher in MHD patients compared with controls, suggesting a severe endothelial dysfunction in these patients [14].

In the present study, we found that predialysis CECs level positively correlated with increased CCA-IMT even after adjusting the confounding effects. An interesting finding in this study was that division of patients into three subgroups based on CCA-IMT showed that greater thickness of CCA-IMT correlated with greater number of circulating endothelial cells. Our findings suggested that higher circulating endothelial cell numbers may be associated with the clinical severity and the progression of carotid atherosclerosis in our patients.

The present study found a positive relationship between circulating endothelial cell numbers and each of BMI and systolic blood pressure in MHD patients before hemodialysis. Obese patients showed elevated serum catecholamine levels and noradrenaline output compared with normal controls [34, 35]. Recent evidences indicate that BMI better reflects body volume and mass, which is closely related to blood pressure [36]. It is clear from previous studies that hypertension is a major risk factor associated with progression of atherosclerosis; thus, direct injury to endothelium can occur through processes, and hypertension-related damage may be a cause of increased numbers of circulating endothelial cells in these patients [37].

Age was positively correlated with both circulating endothelial cell numbers and CCA-IMT in our investigation. Atherosclerotic lesions advance at a variable rate with time; structural and functional alteration of the arterial wall occur with aging [38], which may contribute to the progression of vascular injury. Studies of medicolegal autopsy material in Japan revealed that cerebral atherosclerosis appeared as early as the second decade and increased in severity with age [39], so early detection and prevention or treatment of atherosclerosis are desirable.

The previous study [14] reported that the CECs level of MHD patients with active atherosclerotic cardiovascular disease (ACVD) was more than that of patients without ACVD. In the present study, we did not find the association between predialysis CECs level and primary diseases, and we also did not find any relationship between predialysis CECs level and each of calcium, phosphate, hemoglobin, or albumin in our patients. These results may be partly due to the different selective standard of study population.

## 5. Conclusion

In conclusion, our cross-sectional observation indicated that the number of CECs was closely related to CCA-IMT which is closely linked to severity of carotid atherosclerosis in patients receiving maintenance hemodialysis; the relationship remained the same even if confounding effects were adjusted. The relationship between CECs and CCA-IMT may suggest that CECs could be an important novel marker to the clinical severity of carotid atherosclerosis in this particularly high-risk population. Additional large, long-term longitudinal prospective study is needed to confirm these findings and their clinical relevance.

## Figures and Tables

**Table 1:** Characteristics of patients and controls.

Variable	Patients	Controls	*P* value
Gender (male/female)	62 (29/33)	26 (12/14)	0.958
Age (years)	61.8 ± 12.9	58.3 ± 12.4	0.244
BMI (kg/m^2^)	23.3 ± 3.4	24.1 ± 2.9	0.310
SBP (mmHg)	145.9 ± 18.5	127.4 ± 6.8	**<0.001**
DBP (mmHg)	78.8 ± 10.6	80.2 ± 4.1	0.517
Albumin (g/L)	38.9 ± 3.5	44.2 ± 2.8	**<0.001**
Hemoglobin (g/L)	105.0 ± 16.1	135.9 ± 17.6	**<0.001**
Calcium (mmol/L)	2.1 ± 0.2	2.2 ± 0.1	**0.003**
Phosphate (mmol/L)	1.5 ± 0.5	1.1 ± 0.1	**<0.001**
Total cholesterol (mmol/L)	4.3 ± 1.1	4.9 ± 0.9	**0.016**
Triglycerides (mmol/L)	1.8 ± 1.1	1.6 ± 1.1	0.637
LDL-c (mmol/L)	2.7 ± 0.8	3.4 ± 0.8	**0.001**
HDL-c (mmol/L)	1.0 ± 0.2	1.4 ± 0.3	**<0.001**
Blood urea nitrogen (mmol/L)	22.4 ± 5.7	5.2 ± 1.2	**<0.001**
Serum creatinine (µmol/L)	710.7 ± 231.0	78.8 ± 12.9	**<0.001**

Abbreviations: BMI: body mass index; SBP: systolic blood pressure; DBP: diastolic blood pressure.

**Table 2:** Characteristics of three subgroups in MHD patients.

Variable	All patients	Group A (*n* = 24)	Group B (*n* = 23)	Group C (*n* = 15)
Gender (F/M)	62 (33/29)	24 (13/11)	23 (12/11)	15 (8/7)
Age (y)	61.8 ± 12.9	53.0 ± 11.9	65.6 ± 11.1^a^	70.3 ± 8.4^a^
Dialysis vintage (m)	57.2 ± 45.4	62.4 ± 51.2	62.2 ± 41.3	41.3 ± 40.7
BMI (kg/m^2^)	23.3 ± 3.4	23.1 ± 3.3	23.2 ± 3.3	23.6 ± 4.0
SBP (mmHg)	145.9 ± 18.5	140.1 ± 19.4	148.00 ± 17.1	151.9 ± 17.6
DBP (mmHg)	78.8 ± 10.6	79.9 ± 10.7	78.8 ± 9.8	77.0 ± 12.1
Albumin (g/L)	38.9 ± 3.5	38.8 ± 3.2	39.4 ± 3.5	38.3 ± 3.9
Hemoglobin (g/L)	105.0 ± 16.1	106.3 ± 16.4	103.3 ± 16.3	105.4 ± 16.5
BUN (mmol/L)	22.4 ± 5.7	22.8 ± 6.9	23.3 ± 4.0	20.6 ± 5.9
Scr (µmol/L)	710.7 ± 231.0	794.9 ± 258.5	701.5 ± 171.1	590.1 ± 221.3^a^
CCA-IMT (mm)	0.9 ± 0.4	0.7 ± 0.1	0.9 ± 0.1^a^	1.4 ± 0.4^ac^
CECs (cells/ml)	147.4 ± 95.8	117.0 ± 62.9	135.5 ± 70.6	214.5 ± 138.0^ac^

Note: ^a^ *P* < 0.01: compared with group A;^ c^ *P* < 0.01: compared with group B.

Abbreviations: BMI: body mass index; SBP: systolic blood pressure; DBP: diastolic blood pressure; Scr: serum creatinine; BUN: blood urea nitrogen; CCA-IMT: common carotid artery intima-media thickness; CECs: circulating endothelial cells.

**Table 3:** Association of CCA-IMT with CECs level, age, dialysis vintage, and modality of dialysis adjusted for confounding effects in multiple linear regressions.

	Variable	Beta	95% CI		*t* value	*P* value
			Lower	Upper		
CCA-IMT	CECs	0.461	0.001	0.003	3.684	**0.001**
	Gender	−0.245	−0.331	−0.022	−2.287	**0.026**
	Age	0.385	0.004	0.017	3.391	**0.001**
	Dialysis vintage	−0.035	−0.002	0.002	−0.299	0.766
	Primary disease	0.028	−0.049	0.063	0.256	0.799
	Systolic blood pressure	−0.067	−0.006	0.003	−0.606	0.547
	Body mass index	−0.133	−0.041	0.013	−1.056	0.295

Note: Gender: female 1, male 0; primary disease: chronic glomerulonephritis 1, interstitial nephropathy 2, diabetic nephropathy 3, hypertensive nephrosclerosis 4, and others 5.
